# Regenerative and protective effects of dMSC-sEVs on high-glucose-induced senescent fibroblasts by suppressing RAGE pathway and activating Smad pathway

**DOI:** 10.1186/s13287-020-01681-z

**Published:** 2020-04-29

**Authors:** Xiaowei Bian, Bingmin Li, Jie Yang, Kui Ma, Mengli Sun, Cuiping Zhang, Xiaobing Fu

**Affiliations:** 1 Tianjin Medical University, No. 22, Qixiangtai Road, Heping District, Tianjin, 300070 People’s Republic of China; 2 Research Center for Tissue Repair and Regeneration Affiliated to the Medical Innovation Research Department and 4th Medical Center, PLA General Hospital and PLA Medical College, Beijing, People’s Republic of China; 3 Research Unit of Trauma Care, Tissue Repair and Regeneration, Chinese Academy of Medical Sciences, 2019RU051, Beijing, 100048 People’s Republic of China; 4 Beijing Key Research Laboratory of Skin Injury, Repair and Regeneration, Beijing, People’s Republic of China

**Keywords:** High-glucose, Fibroblasts, Senescence, Small extracellular vesicles, Diabetic wounds

## Abstract

**Background:**

Fibroblasts are crucial for supporting normal wound healing. However, the functional state of these cells is impaired in diabetics because of a high-glucose (HG) microenvironment. Small extracellular vesicles (sEVs) have emerged as a promising tool for skin wound treatment. The aim of this study was to investigate the effects of sEVs derived from human decidua-derived mesenchymal stem cells (dMSC-sEVs) on HG-induced human dermal fibroblast (HDF) senescence and diabetic wound healing and explore the underlying mechanism.

**Methods:**

We first created a HDF senescent model induced by HG in vitro. dMSC-conditioned medium (dMSC-CM) and dMSC-sEVs were collected and applied to treat the HG-induced HDFs. We then examined the proliferation, migration, differentiation, and senescence of these fibroblasts. At the same time, the expressions of RAGE, p21 RAS, Smad2/3, and pSmad2/3 were also analyzed. Furthermore, pSmad2/3 inhibitor (SB431542) was used to block the expression of pSmad2/3 to determine whether dMSC-sEVs improved HDF senescence by activating Smad pathway. Finally, we assessed the effect of dMSC-sEVs on diabetic wound healing.

**Results:**

The HG microenvironment impaired the proliferation, migration, and differentiation abilities of the HDFs and accelerated their senescence. dMSC-CM containing sEVs improved the proliferation and migration abilities of the HG-induced fibroblasts. dMSC-sEVs internalized by HG-induced HDFs not only significantly promoted HDF proliferation, migration, and differentiation, but also improved the senescent state. Furthermore, dMSC-sEVs inhibited the expression of RAGE and stimulated the activation of Smad signaling pathway in these cells. However, SB431542 (pSmad2/3 inhibitor) could partially alleviate the anti-senescent effects of dMSC-sEVs on HG-induced HDFs. Moreover, the local application of dMSC-sEVs accelerated collagen deposition and led to enhanced wound healing in diabetic mice. The detection of PCNA, CXCR4, α-SMA, and p21 showed that dMSC-sEVs could enhance HDF proliferation, migration, and differentiation abilities and improve HDF senescent state in vivo.

**Conclusion:**

dMSC-sEVs have regenerative and protective effects on HG-induced senescent fibroblasts by suppressing RAGE pathway and activating Smad pathway, thereby accelerating diabetic wound healing. This indicates that dMSC-sEVs may be a promising candidate for diabetic wound treatment.

## Introduction

Diabetic chronic wounds, as a common complication of diabetes, refer to wounds that cannot attain anatomical and functional wound healing standards after regular treatment for 4 weeks or more [1]. In China, 25% of patients with diabetes develop a diabetic foot ulcer, which burdens both individuals and their countries [2]. There are various treatments for diabetic ulcers, including removal of necrotic tissue from the wound (debridement), reduction of pressure in the wound (offloading), and surgical revascularization, among others. However, in many cases, these therapies are ineffective, which increases the risk for limb amputation. Therefore, enormous effort has been invested to develop innovative and efficient treatments for diabetic nonhealing wounds.

During wound healing, HDFs migrate to the wound bed to proliferate and to participate in synthesizing and secreting the extracellular matrix (ECM) as well as expressing cytokines and growth factors [3]. More importantly, HDFs differentiate into myofibroblasts, enhancing wound contraction, thereby promoting wound closure [4]. However, these capacities are impaired in diabetic microenvironments [5]. The mechanism for this impairment includes cellular senescence induced by excessive oxidative stress. Advanced glycation end products (AGEs) are a form of covalent compounds produced by the oxidation reaction of glucose and protein or lipid under non-enzymatic conditions. AGEs and their receptor RAGE play a crucial role in diabetes [6]. AGEs in vivo can bind to RAGE and generate reactive oxygen species (ROS). ROS accelerate the shortening of the telomere length of endothelial cells [7]. Therefore, we hypothesized that ROS may be pathogenically linked with the impaired and senescent state of HDFs.

sEVs, defined as 50–150-nm-sized vesicles, which are secreted from cells, were discovered more than 30 years ago. sEVs are thought to be carriers of intercellular biological information, because they may contain nucleic acids, lipids, and proteins, thereby playing an indispensable role in cell-to-cell communication [8]. Furthermore, the composition of sEVs varies according to their origin, and thus, the information they carry also differs [9]. Biological characteristics and functions of sEVs suggest their potential application for cell-free regeneration strategies, which may avoid the disadvantages of current stem cell transplantation techniques. It has been proposed that mesenchymal stem cell-derived exosomes are effective in reducing the damage of HaCaT cells exposed to hydrogen peroxide [10]. However, there is a lack of studies on the diabetic wound reparative potential of decidua-derived mesenchymal stem cells (dMSCs) isolated from the human placenta, which are an attractive source of transplantable stem cells for wound repair, because they pose no risk to donors, have easy accessibility, and display a low incidence of graft-versus-host disease [11]. Komaki et al. found that sEVs isolated from human placenta mesenchymal stem cells (PMSCs) enhanced endothelial tube formation [12]. Further study demonstrated that sEVs released from PMSCs by NO stimulation revealed superior angiogenic effects and ameliorated limb function in a murine model of hind limb ischemia [13]. Whether dMSC-sEVs can ameliorate diabetic chronic wounds is not fully understood, and the potential mechanisms need to be further elucidated.

In the present study, we observed the effects of dMSC-sEVs on the proliferation, migration, differentiation, and senescence of HDFs and explored the underlying mechanism. Moreover, the therapeutic effect of dMSC-sEVs on diabetic wound healing was evaluated.

## Methods

### Ethics statement

Tissues were obtained from human subjects after they gave their informed consent. The protocol was approved by the national ethics committee in China.

### Cell isolation and identification

Human placentas’ dMSCs were obtained from healthy mothers during routine Caesarian section births. The external membranes were removed from the placenta, then we cut the maternal side (decidua basalis) of the placenta into 1 mm^3^ pieces and put them into culture dishes containing with Dulbecco’s modified Eagle’s medium/F12 (DMEM/F12, Gibco, USA), 100 U/mL collagenase type I (Sigma-Aldrich, Germany), and 5 μg/mL DNase I (Solarbio, China). Culture dishes were incubated in a cell incubator (37 °C, 5% CO_2_, 2 h), and then we aspirated the supernatant into a centrifuge tube and centrifuged (1000 rpm, 5 min). After centrifugation, the supernatant was discarded. The pellets were resuspended in DMEM/F12 contained 10% fetal bovine serum (FBS), seeded in culture dishes, and incubated overnight.

After 3–5 passages, the flow cytometer was used to identify cell surface markers of dMSCs. The dMSCs were washed and incubated with fluorescence-conjugated antibodies (Abcam, USA) (CD105-FITC, CD90-FITC, CD73-PE, CD19-FITC, CD45-FITC, CD34-FITC, and HLA-DR-FITC) at room temperature for 45 min. For multi-differentiation of dMSCs, cells were incubated with osteogenic differentiation medium (Cyagen, China), adipogenic differentiation medium (Cyagen, China), and chondrogenic differentiation medium (Cyagen, China), respectively. Then, the induced cells were stained separately with Alizarin Red S, Oil Red O, and Alcian blue to assess osteogenic, adipogenic, and chondrogenic differentiation. The cell images were captured by using an Olympus IX71 light microscope (Olympus, Japan).

HDFs were isolated using previously described protocols [14]. HDFs were incubated with glucose at the final concentrations of 5.5 mM (NG), 26 mM (HG1), and 35 mM (HG2) for 1, 3, 5, and 7 days. Then the 5th cells were used for the cell proliferation assay, scratch assay, ROS generation evaluation, and senescence-associated β-galactosidase staining (SA-β-gal staining).

### Collection of dMSC-conditioned medium

dMSC-CM was collected from the supernatant of a high dMSC-density culture. After 3–5 passages, the culture medium was changed into DMEM/F12 containing 10% exosome-free FBS (SBI, USA). To investigate whether dMSC-CM containing sEVs has effects on HG-induced HDFs, the dMSCs were pretreated with or without 2.5 μM GW4869 (Sigma-Aldrich, Germany) for 12 h, and then we collected the culture medium. Both two kinds of dMSC-CM were stored in − 80 °C until use.

### Isolation and characteristics of dMSC-sEVs

dMSC-CM was collected every 48 h starting at passage 3 until passage 7. dMSC-sEVs were pooled separately from different patients. But in the same experiment, the dMSC-sEVs were from the same patient. dMSC-sEVs were extracted from the dMSC-CM by using a series of high-speed centrifugation steps with the protocol described by Théry et al. [15]. The characteristics of dMSC-sEVs were performed by transmission electron microscope (TEM) (Hitachi, Japan) and nanoparticle tracking analyzer (Particle Metrix GmbH, Germany). Expressions of the sEV markers including CD9, CD63, CD81, TSG101, and glucose-regulated protein 94 (Grp94) (Abcam, USA) were analyzed by Western blot. The aged HDFs were treated with dMSC-sEVs at different concentrations (1.74 × 10^11^ particles/mL, 3.48 × 10^11^ particles/mL, 5.22 × 10^11^ particles/mL, and 6.92 × 10^11^ particles/mL) for the cell proliferation assay, cell cycle, scratch assay, ROS generation evaluation, and SA-β-gal staining. At the same time, the expressions of α-SMA, collagen I, RAGE, p21 RAS, Smad2/3, and pSmad2/3 (Abcam, USA) were also analyzed by Western blot. To determine whether dMSC-sEVs improved HDF senescence by activating Smad signaling pathway, HG-induced HDFs were cultured with pSmad2/3 inhibitor (SB431542, 10 μM, MedChemExpress, USA) and dMSC-sEVs (5.22 × 10^11^ particles/mL) for 48 h. Then these cells were harvested for the detection of pSmad2/3 by Western blot and SA-β-gal by SA-β-gal staining kit.

### Uptake assay of dMSC-sEVs

dMSC-sEVs were labeled with PKH67 (Sigma-Aldrich, Germany) according to the manufacturer’s protocol. The internalization of dMSC-sEVs by HDFs was counterstained with Phalloidin- Rhodamine B (cytoskeleton) and Dapi (cell nucleus) and observed under confocal microscope (Leica, Germany).

### Cell proliferation and cell cycle assay

The proliferation ability of HDFs was measured with a cell counting kit-8 (CCK-8, Dojindo Molecular Technologies, Japan) as previously described [16]. Briefly, HDFs were seeded in 96-well plates with 8 × 10^3^ cells/well and cultured with HG, dMSC-CM, or dMSC-sEVs. When it reached the time point, the cells were incubated for 3 h with CCK-8 regent (100 μL, 10%). The plates were read on an enzyme immunoassay analyzer (Bio-Rad 680, Hercules, USA) at 450 nm. For cell cycle assay, the cells cultured in different medium were collected and fixed in 70% cold ethanol at 4 °C overnight. The next day, single-cell suspensions were digested with 100 μL DNase-free RNase in 37 °C cell incubator, and then we added 400 μL propidium iodide (PI) solution for DNA staining (1 h, 4 °C). The PI fluorescence and forward light scattering were detected with a flow cytometer (BD FACS Calibur™, Becton-Dickinson, USA). The percentage of cells in every phase was calculated.

### Scratch assay

The migration property was evaluated by scratch assay. HDFs were cultured in a six-well plate and when 90% confluence was reached, the cells were scratched with a pipet tip through the well bottom center. At design time, the images were taken using an inverted microscope (Leica DMI 3000B, Solms, Germany). Distances between two sides of the scratch were measured by ImageJ software. The migration rate of the scratch was calculated as follows: migration rate (%) = (*A*_0_ − *A*_*t*_)/*A*_0_ × 100, where *A*_0_ represents the initial scratch distances and *A*_*t*_ was defined as the remaining scratch distances at the measured time point.

### ROS generation evaluation

After cultured under design condition, HDFs were washed with phosphate buffer saline (PBS) and incubated with 10 μM 2′,7′-dichlorodihydrofluorescein diacetate (DCFH-DA, Sigma-Aldrich, Germany) in a cell incubator (37 °C, 5% CO_2_, 30 min). The cells were incubated with 100 mM Rosup as positive control and the probe was omitted as negative control. The accumulation of ROS in cells was viewed on a fluorescence microscope and imaged (Leica DMI 3000B, Solms, Germany).

### SA-β-gal staining

SA-β-gal staining was performed with a SA-β-gal staining kit (Sigma-Aldrich, Germany) according to the manufacturer’s instructions to evaluate the SA-β-gal expression in HDFs. HDFs were washed three times with PBS and fixed with 4% paraformaldehyde for 30 min. After incubated with staining solution overnight under 37 °C CO_2_-free circumstance, the cells were observed under an inverted phase contrast microscope (Leica DMI 3000B, Solms, Germany). The ratio of SA-β-gal-positive cells was determined by counting the blue cells versus total cells.

### Western blot

The total protein was extracted using RIPA buffer with a total protease phosphatase inhibitor mix (Solarbio, China). Protein extracts were separated on a 10% SDS-PAGE, transferred to polyvinylidene fluoride membranes, and blocked with 5% non-fat dried milk in TBST. The membranes were incubated with primary antibodies including anti-CD9, anti-CD63, anti-CD81, anti-TSG101, anti-Grp94, anti-RAGE, anti-p21 RAS, anti-phosphorylate Smad2/3 (anti-pSmad2/3), anti-Smad2/3, anti-α-SMA, anti-collagen I, and anti-p21 (Abcam, USA) at 4 °C overnight, followed by the incubation with horseradish peroxidase-conjugated goat anti-rabbit secondary antibody (ZSGB-BIO, China). The immunoreactive bands were developed using an ECL kit (Solarbio, China) and exposure was performed with the UVITEC Alliance MINI HD9 system (UVITEC, Britain).

### Animal experiments

All procedures were guided by the Animal Research Committee of Chinese PLA General Hospital. Forty female diabetic mice (BKS-Dock Lepr^em2Cd479^, db/db) were used in this experiment. After shaving the back of the mice, 16 mm diameter full-thickness excisional wounds were created on the back. Afterward, all mice were randomly assigned into PBS groups and dMSC-sEV groups. dMSC-sEVs (100 μL, 5.22 × 10^11^ particles/mL) and PBS (100 μL) were injected around the wounds at 4 sites (25 μL per site) at 7, 14, 21, and 28 days [14, 17]. dMSC-sEV concentration was selected based on the results of the preliminary experiment. There were five mice for each time point. Wound closure rate was calculated using the equation: wound closure rate (%) = 100 × (original wound area − actual wound area)/original wound area.

### Immunofluorescence staining

The sections from the wounds were deparaffinized in xylene and rehydrated in graded ethanol. After 70 °C water bath with citrate repair solution (pH = 6.0), the sections were incubated with 5% goat serum for 2 h, and then with primary mouse monoclonal anti-mouse anti-PCNA (1:200, Abcam, USA), anti-α-SMA (1:200, Abcam, USA) and rabbit monoclonal anti-mouse CXCR4 (1:200, Abcam, USA), anti-p21 (1:800, Abcam, USA) overnight at 4 °C. After that, the sections were washed three times with PBS and then incubated with rhodamine-labeled goat anti-rabbit IgG secondary antibody (1:100, Zsbio, China) and FITC-labeled goat anti-mouse IgG secondary antibody (1:100, Zsbio, China) for 2 h at room temperature. Nuclei were stained with DAPI (Thermo, USA) and fluorescence images were visualized with confocal microscope (Leica, Germany).

### Statistical analysis

All the results were expressed as the mean ± SEM. Comparisons between the two groups were evaluated with the unpaired Student’s *t* test. For more than two groups, one-way or two-way ANOVA with Bonferroni post hoc test was used. The value of *p* < 0.05 was considered statistically significant. Statistical analysis was conducted using GraphPad Prism 8.0 software.

## Results

### HG impaired the proliferation and migration abilities of HDFs

**Fig. 1:**
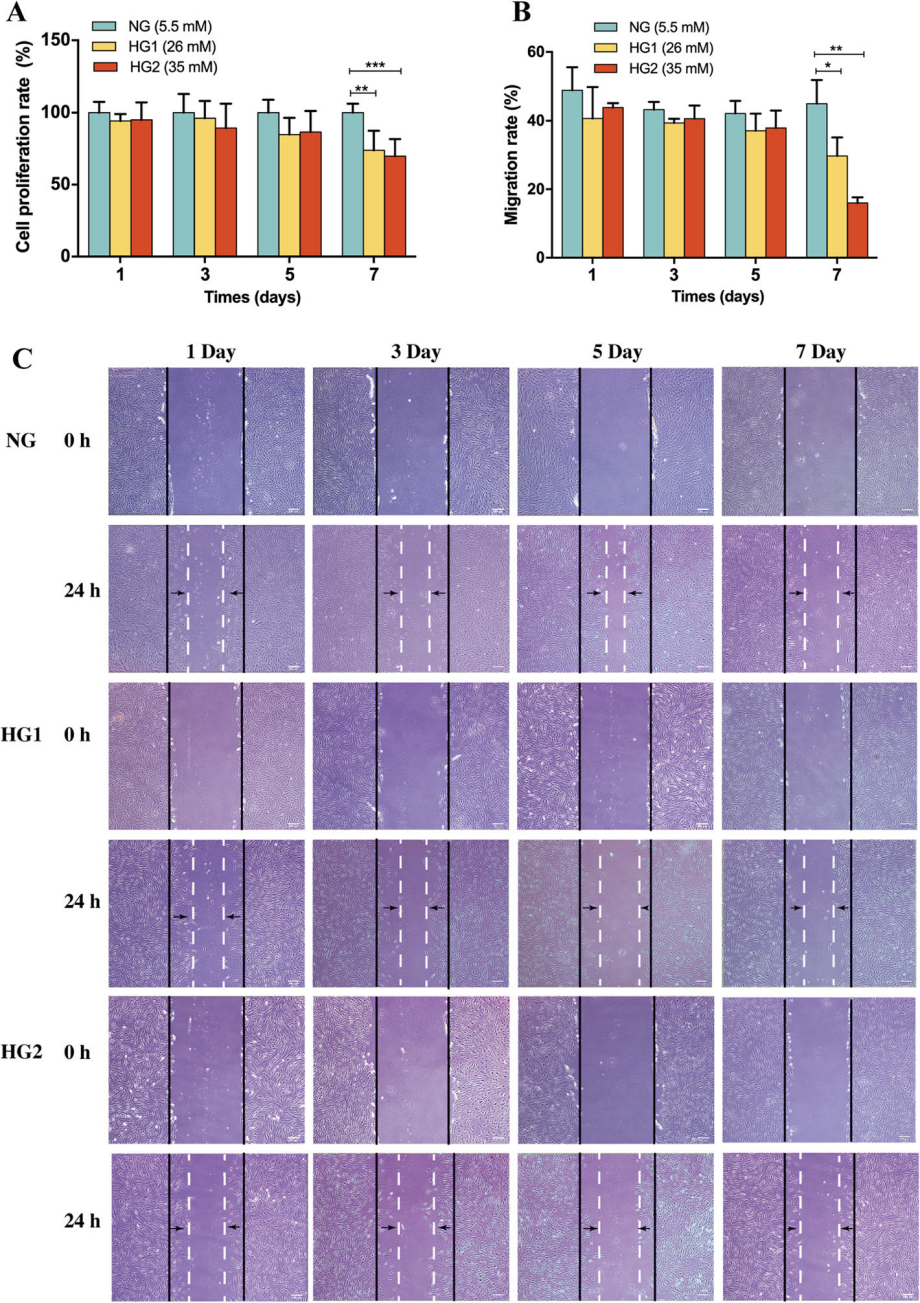
HG impaired HDF proliferation and migration. **a** After incubation of HDFs with HG for 1, 3, 5, and 7 days, the cell proliferation rate was evaluated using the CCK8 assay. **b**, **c** Fibroblast migration was determined using the in vitro wound closure assay. Scale bar = 200 μm. The HDFs cultured in NG were the control group (*n* = 5). *p < 0.05, **p < 0.01, ***p < 0.001

To investigate whether HG regulated the proliferation and migration of HDFs, the CCK-8 and scratch assays were performed, respectively, for HDFs with different glucose concentrations. After 7 days of HG treatment, HG1 and HG2 significantly reduced the cell proliferation rate compared with NG [HG1 (73.89 ± 13.57%) and HG2 (69.86 ± 11.8%) versus NG (100 ± 6.29%), *p* < 0.01 and *p* < 0.001, respectively] (Fig. 1a). Furthermore, at day 7, the migration rate of the HG1 and HG2 group were significantly decreased compared with the NG group [HG1 (29.74 ± 5.64%) and HG2 (16.02 ± 1.64%) versus NG (45.03 ± 6.76%), *p* < 0.05 and *p* < 0.01, respectively] (Fig. 1b, c). These results demonstrated that the proliferation and motility capacities of HDFs were significantly suppressed by prolonged stimulation with HG.

### HG accelerated HDFs senescence

**Fig. 2:**
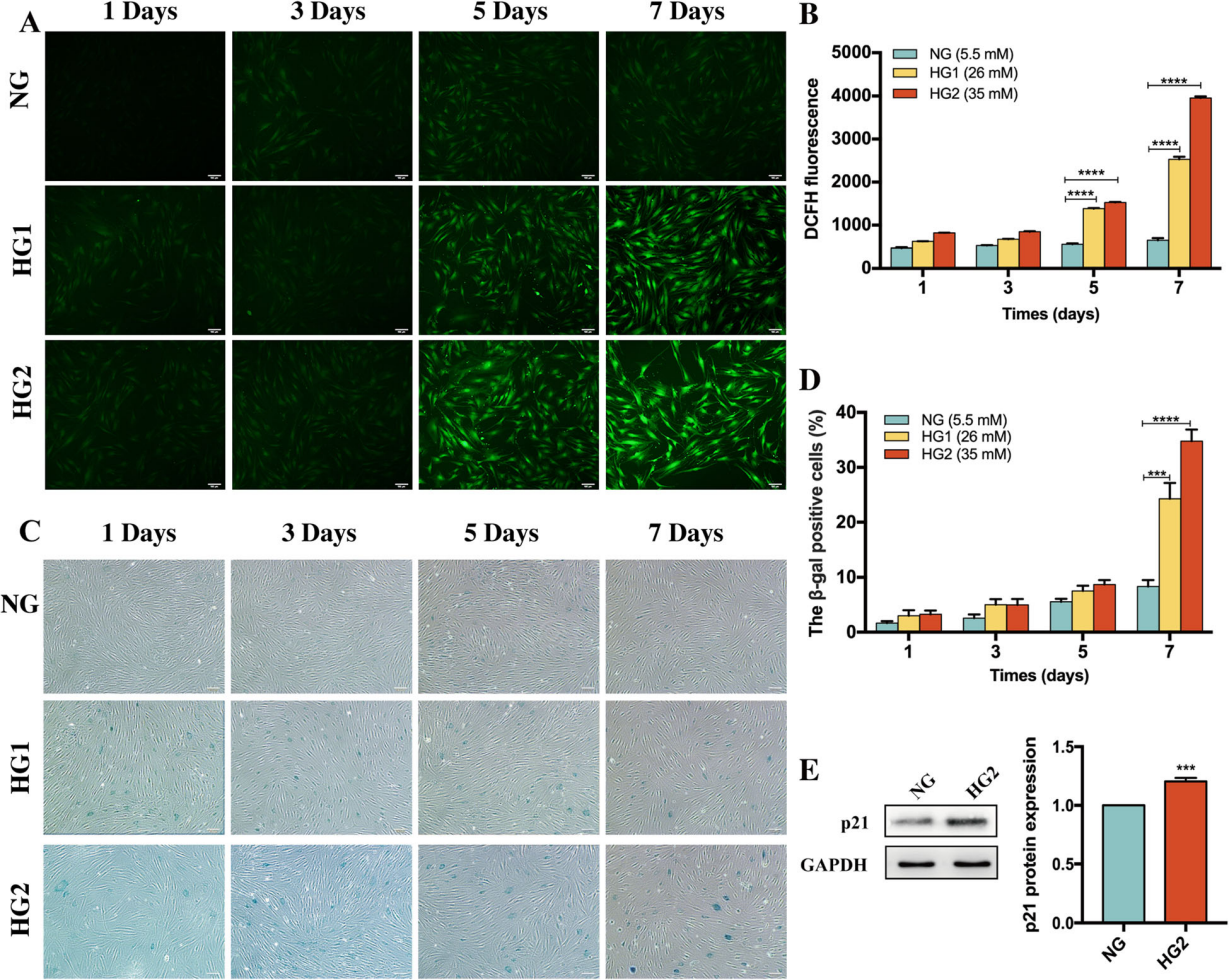
HG accelerated fibroblast senescence. **a** HG-induced ROS generation in DFs. HDFs were treated with HG for 1, 3, 5, and 7 days, and then the cells were labeled with DCFH-DA, followed by fluorescence microscopy. Scale bar = 100 μm. **b** ROS generation in HDFs labeled with DCFH-DA and analyzed by flow cytometer. The fluorescence intensity is expressed as arbitrary units (a.u.) (*n* = 5). **c**, **d** SA-β-gal-kit was adopted to evaluate the SA-β-gal assay in HDFs (*n* = 5). Scale bar = 100 μm. **e** The expression level of p21 and the quantification result. The results were normalized to GAPDH expression (*n* = 5). ***p < 0.001, ****p < 0.0001

Increased intracellular ROS generation is a marker of cell senescence [18]. HDFs were treated with NG, HG1, or HG2 for 1, 3, 5, or 7 days, and cellular ROS generation was determined by DCFH-DA fluorescence (Fig. 2a, b). The results showed that there was no significant difference between the HG1 or HG2 groups compared with the NG group at 1 or 3 days. However, ROS generation was gradually increased after exposure to HG1 or HG2 for 5 days [HG1 (1426 ± 65.39 and HG2 (1606 ± 65.39) versus NG (566.7 ± 23.09), *p* < 0.0001] and 7 days [HG1 (2901 ± 358.3) and HG2 (4021 ± 154.9) versus NG (650 ± 50), *p* < 0.0001]. The results showed that the intracellular ROS levels increased with increasing glucose concentration and prolonged culture time, reaching a maximum level with HG2 at day 7 as compared with the lower glucose concentrations and fewer days of exposure to HG. Cellular senescence is associated with the increased expression levels of SA-β-gal and P21 (cyclin-dependent kinase inhibitors). SA-β-gal is a typical marker expressed in aged HDFs [19]. At days 1, 3, and 5 after HG stimulation, the SA-β-gal expression was not significantly different compared with the NG group (Fig. 2c, d). However, by day 7, the SA-β-gal expression in the treatment groups HG1 and HG2 was significantly higher (24.29 ± 2.86% and 34.81 ± 2.12%, respectively) than in the NG group (8.33 ± 1.16%) (*p* < 0.001 and *p* < 0.0001, respectively). These results were consistent with previous research [20]. Therefore, HDFs incubated with HG2 for 7 days were used in the following experiments.

Previous studies reported that p21 is a representative molecular effector as well as marker for cellular senescence [21]. In agreement with previous findings [20, 22], the Western blot results showed that p21 expression was significantly upregulated (1.20 ± 0.03-fold, *p* < 0.001) by incubating with HG2 for 7 days compared with the NG group (Fig. 2e). These results suggested that HDFs exhibit senescent behavior under HG2 stimulation for at least 7 days.

### Characterization of dMSCs

**Fig. 3:**
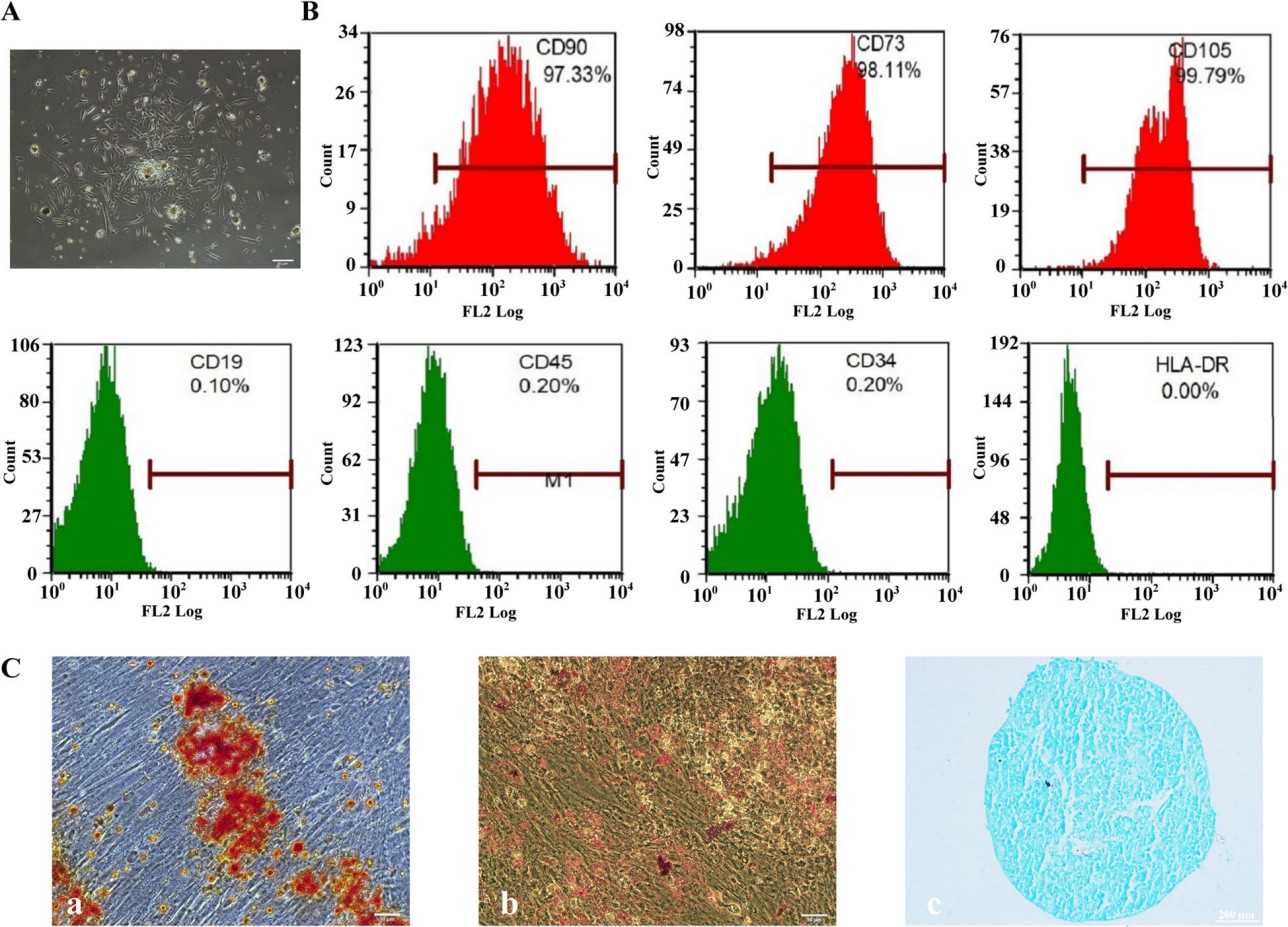
Characteristics of dMSCs. **A** Bright-field microscope image of colonies formed by dMSCs. Scale bar = 50 μm. **B** Flow cytometric analysis of cell surface markers. **C** (a) Calcification assessed by Alizarin Red S staining of dMSCs. **C** (b) Lipid droplet formation stained by Oil Red in dMSCs after adipogenic induction. Scale bar = 50 μm. **C** (c) Chondrogenic differentiation of dMSCs assessed by Alcian blue stain. Scale bar = 200 μm

Cell colonies appeared 5–7 days after initial plating. The cells exhibited a spindle shape (Fig. 3A). At 3–5 passages, flow cytometry analysis showed that the dMSCs were highly positive for MSC surface markers, including CD90 (97.33%), CD73 (98.11%), and CD105 (99.97%), but negative for CD19 (0.10%), CD45 (0.20%), CD34 (0.20%), or HLA-DR (0.00%) (Fig. 3B). All these features were consistent with the findings of previous studies [23, 24]. When cultured in osteogenic, adipogenic, or chondrogenic medium, dMSCs were able to differentiate into osteoblasts, adipocytes, and chondroblasts, respectively, as evidenced by Alizarin Red S staining (Fig. 3C, a), Oil Red O staining (Fig. 3C, b), and Alcian blue staining (Fig. 3C, c), respectively. These results suggest that we have isolated dMSCs successfully.

### dMSC-CM improved proliferation and migration abilities of HDFs

**Fig. 4:**
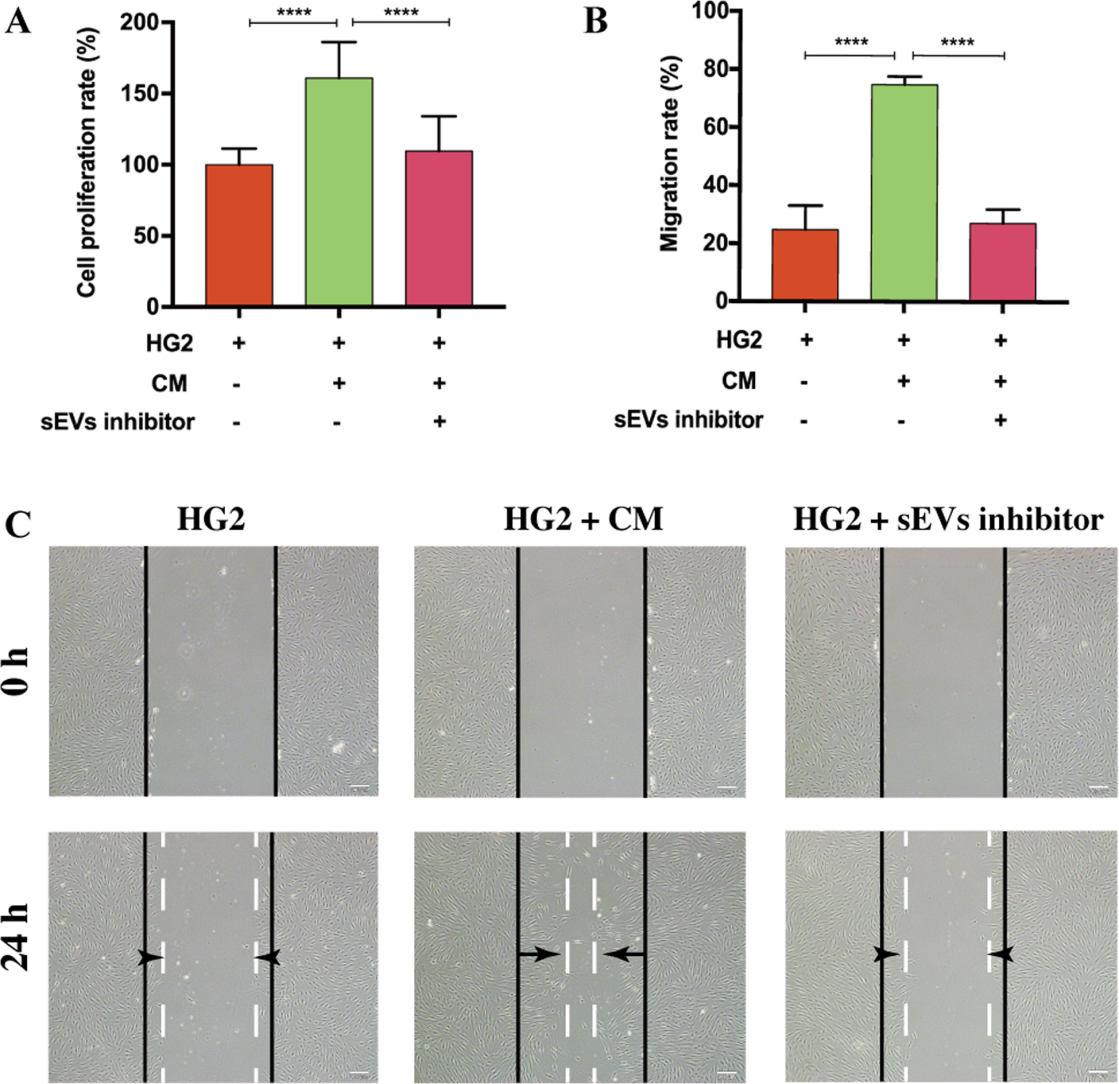
dMSC-CM improved the proliferation and migration abilities of HDFs. **a** After incubation of HG-induced HDFs with dMSC-CM with or without GW4869, cell proliferation rate was evaluated using the CCK8 assay (*n* = 5). **b**, **c** Cell migration capability was assessed by scratch assay (*n* = 5). Scale bar = 200 μm. ****p < 0.0001

To elucidate whether dMSC-CM containing sEVs improves the proliferation and migration abilities of HDFs, dMSC-CM was collected and used to treat HG-induced HDFs. The cell proliferation rate of HG-induced HDFs was significantly elevated after cultured with dMSC-CM (160.8 ± 25.3%) (*p* < 0.0001), but no significant difference was observed on the addition of sEVs blocker GW4869 (109.7 ± 24.43%) (Fig. 4a). Moreover, cell migration capability increased in HDFs stimulated with dMSC-CM (74.82 ± 2.77%) (*p* < 0.0001), but the effect decreased when GW4869 was added into dMSC-CM (27.12 ± 4.81%) (*p* < 0.0001) (Fig. 4b, c). Therefore, we speculated that dMSC-sEVs in dMSC-CM probably enhance the proliferation and migration abilities of HDFs.

### Characterization of dMSC-sEVs

**Fig. 5:**
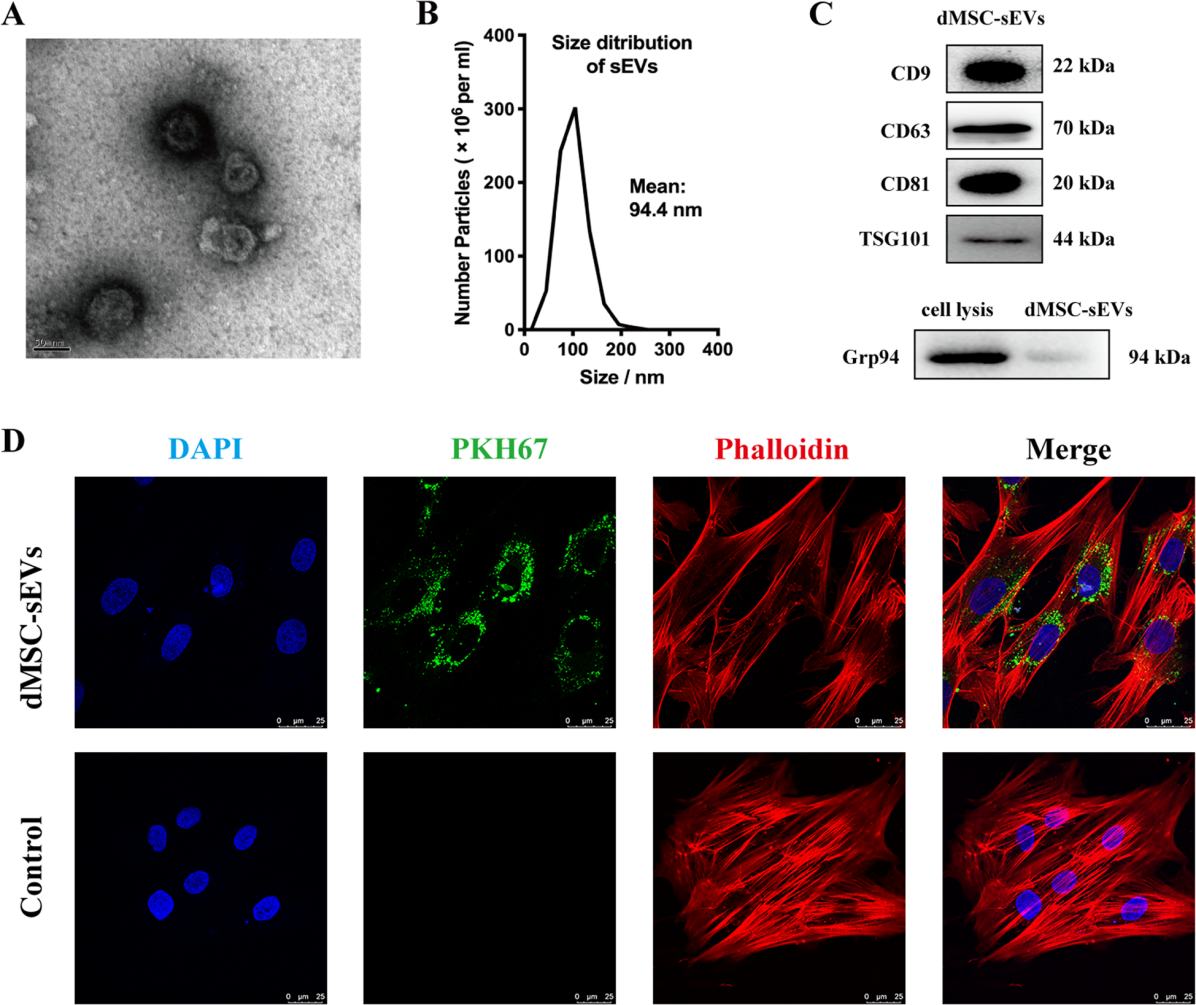
Characterization of dMSC-sEVs. **a** Ultrastructure of dMSC-sEVs, scale bar = 50 nm. **b** Dynamic tracking capture and particle size distribution were measured by nanoparticle tracking analyzer. **c** The expression level of CD9, CD63, CD81, TSG101, and Grp94. **d** Internalization assay of dMSCs-sEVs by HDFs. dMSC-sEVs were marked by the green fluorescence (PKH-67) and cytoskeleton were marked by red fluorescence (phalloidin). Scale bar = 25 μm

The ultrastructure of the dMSC-sEVs was presented in Fig. 5a, with a diameter of approximately 100 nm and being cup shaped. The particle size distribution was determined using a nanoparticle tracking analyzer (NTA) (Fig. 5b). The results showed that the size of 90% particles were distributed between 63.8 and 125 nm (mean diameter = 94.4 nm), similar to a previous description of sEVs [12]. sEV markers including CD9, CD63, CD81, and TSG101 were expressed by the dMSC-sEVs (Fig. 5c). Grp94, as a negative protein marker of sEVs [25], was not detectable in our study. The green fluorescent dye (PKH-67-labeled dMSC-sEVs) was transferred into the cytoskeleton (red fluorescent dye phalloidin-labeled) after 12 h (Fig. 5d).

### dMSC-sEVs improved proliferation, migration, and differentiation abilities of HDFs

**Fig. 6:**
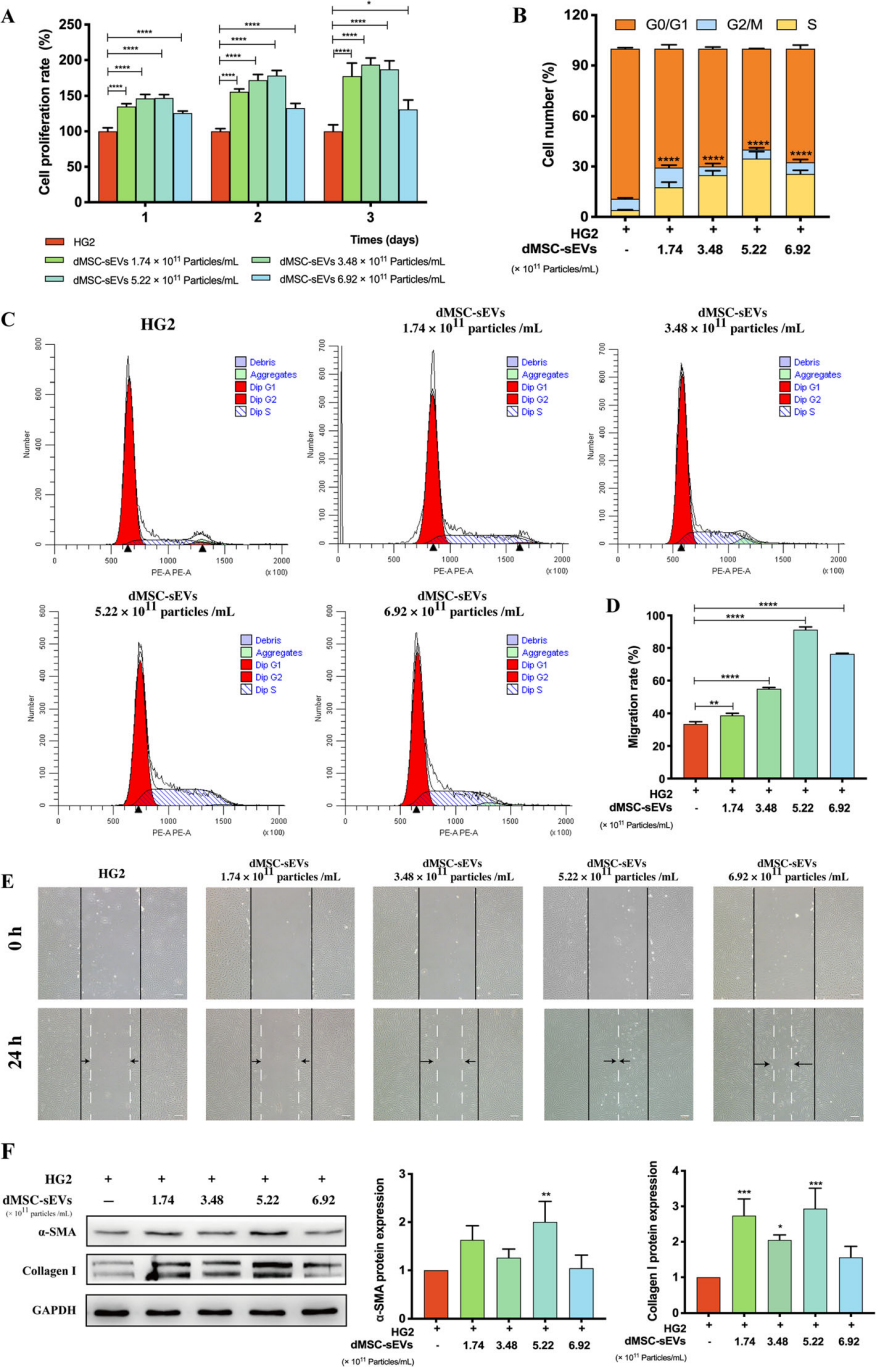
dMSC-sEVs improved the proliferation, migration, and differentiation abilities of fibroblasts. **a** CCK-8 analysis was applied to measure the effect of dMSC-sEVs on the proliferation of HG aged HDFs (*n* = 5). **b**, **c** Effects of the dMSC-sEVs on the cell cycle progression of HG aged HDFs (*n* = 5). **d**, **e** The migration ability has been determined by migration assay (*n* = 5). Scale bar = 200 μm. **f** The expression level of α-SMA and collagen I and the quantification results. The results were normalized to GAPDH expression (*n* = 5). *p < 0.05, **p < 0.01, ***p < 0.001, ****p < 0.0001

CCK-8 analysis was applied to determine whether dMSC-sEVs could reverse the inhibitory effects of HG on HDF proliferation. The results showed that the proliferation rate significantly increased under the stimulation of different dMSC-sEV concentrations (Fig. 6a). Furthermore, over the concentration range from 1.74 × 10^11^ particles/mL to 5.22 × 10^11^ particles/mL, the promotion effect was linearly related to the dMSC-sEV concentration. But at the concentration of 6.92 × 10^11^ particles/mL, the therapeutic effect slightly decreased. The possible reason may due to co-isolated protein contaminants which at high concentration interfere the therapeutic effects of dMSC-sEVs.

To investigate the effect of dMSC-sEVs on the cell cycles of aged HDFs, three cell subpopulations (G_0_/G_1_, S, and G_2_/M) were evaluated from the DNA distribution of the cells determined by flow cytometry. The results indicated that after treatment with dMSC-sEVs, the proportion of HDFs in S and G2/M was increased markedly compared with the HG2 group, indicating high cell proliferative abilities of the HG-induced cells treated with dMSC-sEVs (Fig. 6b, c). Moreover, we observed that dMSC-sEVs at the concentration of 5.22 × 10^11^ particles/mL had the most obvious effect compared with other concentrations.

Consistent with the effects of dMSC-sEVs on proliferation of HDFs, the results of scratch assay demonstrated that the migration rate of HDFs treated with dMSC-sEVs was increased significantly compared with the HG2 group (HG2 group, 33.55 ± 1.41%; 1.74 × 10^11^ particles/mL group, 38.75 ± 1.37%; 3.48 × 10^11^ particles/mL group, 55.11 ± 0.86%; 5.22 × 10^11^ particles/mL group, 91.25 ± 1.64%; 6.92 × 10^11^ particles/mL group, 76.34 ± 0.52%) and dMSC-sEVs at the concentration of 5.22 × 10^11^ particles/mL displayed the strongest stimulation of migration.

Subsequently, we investigated the effect of dMSC-sEVs on the differentiation of HG-induced HDFs. Numerous studies have demonstrated that α-SMA and collagen I are myofibroblast markers. In our study, we found that α-SMA and collagen I protein expression was significantly increased in dMSC-sEVs groups compared with the HG2 group (Fig. 6f), especially at the concentration of 5.22 × 10^11^ particles/mL. The data suggest that dMSC-sEVs promote the differentiation of HG-induced HDFs into myofibroblasts.

### dMSC-sEVs improved HDF senescence

**Fig. 7:**
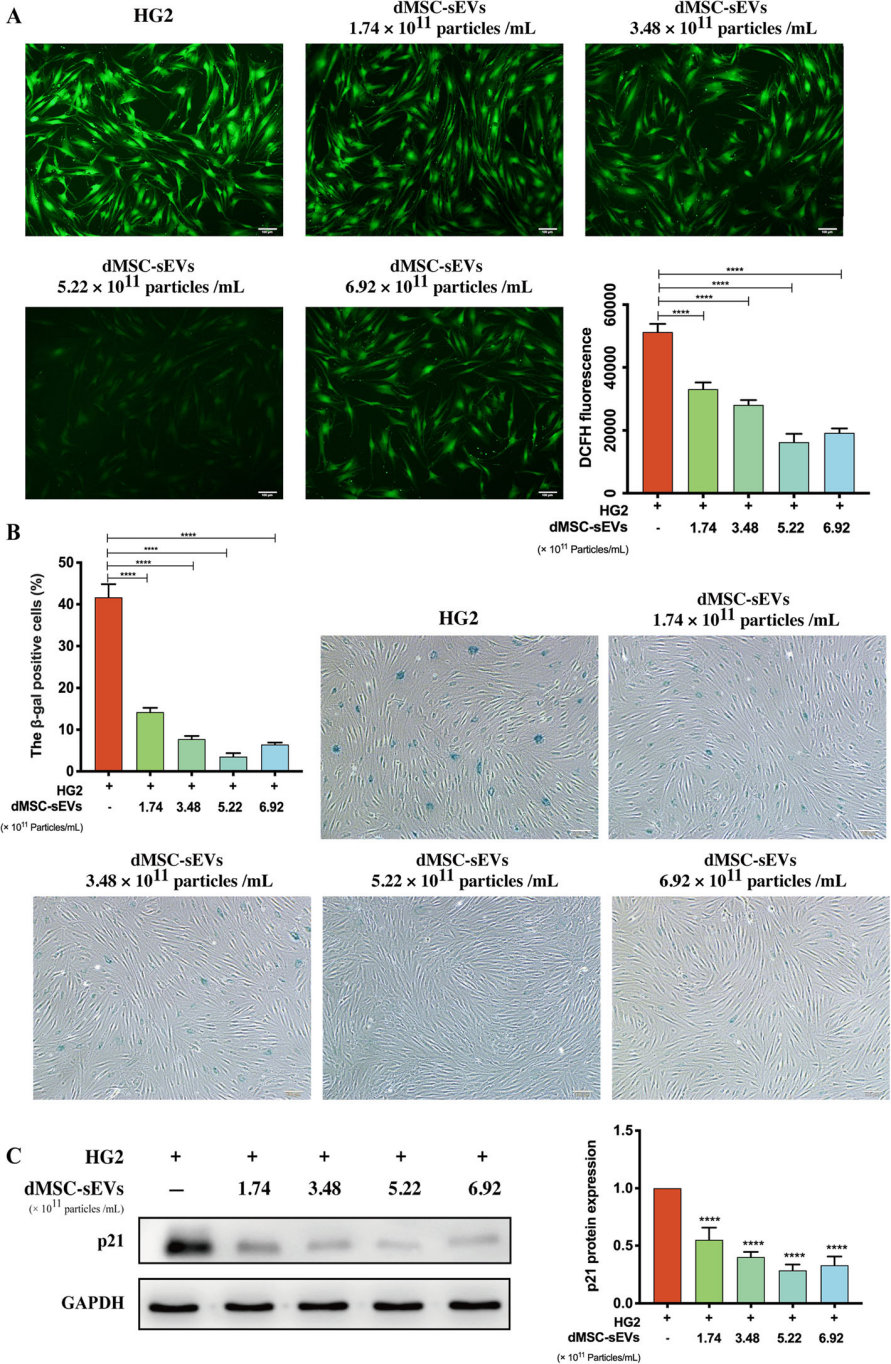
dMSC-sEVs improved fibroblast senescence. **a** ROS generation in HG aged HDFs with or without dMSC-sEVs. The fluorescence intensity is expressed as arbitrary units (a.u.) (*n* = 5); scale bar = 100 μm. **b** SA-β-gal assay was performed on HDFs after being treated with different concentrations of dMSC-sEVs. SA-β-gal-staining cells were counted in five randomized fields. **c** The expression level of p21 and the quantification result. The results were normalized to GAPDH expression (*n* = 5). *p < 0.05, **p < 0.01, ***p < 0.001, ****p < 0.0001

As a strong oxidizing agent, ROS is one of the direct causes to cellular senescence. Figure 7a presents the intracellular ROS generation in HDFs. As expected, extensive fluorescence was observed in the HG aged cells, but in the dMSC-sEV group (5.22 × 10^11^ particles/mL), fluorescence intensity was decreased significantly, representing the inhibition of ROS generation. To further verify the effect of dMSC-sEVs on cell senescence, SA-β-gal assay was performed on HG aged HDFs with or without dMSC-sEVs. Consistent with the change of ROS generation, the expression of SA-β-gal in HG aged cells was blocked by dMSC-sEVs (Fig. 7b). We also detected the expression of senescent marker p21 in every group (Fig. 7c). The p21 level in dMSC-sEV groups was significantly reduced compared with the HG2 group (*p* < 0.0001). These results suggested that dMSC-sEVs significantly protected HDFs against cellular senescence induced by HG.

### dMSC-sEVs suppressed RAGE pathways and activated Smad pathways

**Fig. 8:**
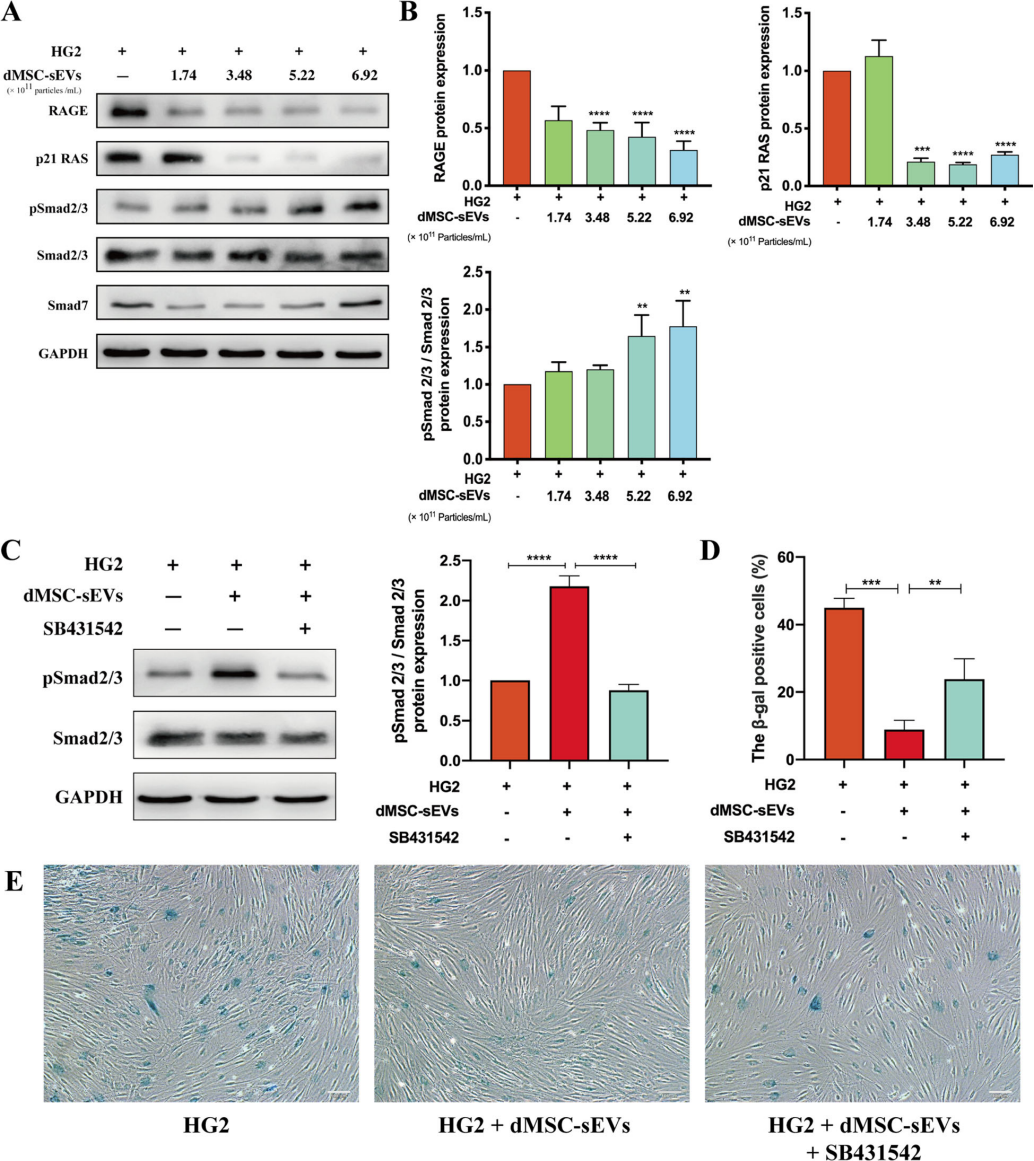
dMSC-sEVs suppressed RAGE pathway and activated Smad pathway. **a**, **b** The expression level of RAGE, p21 RAS, and pSmad2/3 in HG aged HDFs with or without dMSC-sEVs. **c** The expression level of pSmad2/3 in HG aged HDFs treated with dMSC-sEVs or SB431542. The results were normalized to GAPDH expression (*n* = 5). **d**, **e** SA-β-gal expression in HG aged HDFs treated with dMSC-sEVs or SB431542. *p < 0.05, **p < 0.01, ***p < 0.001, ****p < 0.0001

In recent years, AGEs have been linked to impaired diabetic wound healing [26], and they may play a role in its pathogenesis [27]. Other investigators have reported that blockade of RAGE using the recombinant soluble form of RAGE, the extracellular ligand-binding domain of the receptor, restores effective wound closure in diabetic mice [28]. In the present study, the expressions of RAGE and its downstream protein p21 RAS were decreased significantly in dMSC-sEV groups compared with that in HG2 group (Fig. 8a, b). Smad signaling has been shown to be the principal signaling pathway for HDFs in wound healing. The pSmad2/3 level was significantly elevated in dMSC-sEV-treated groups compared with that observed in the HG2 group (Fig. 8a, b). In the presence of pSmad2/3 inhibitor SB431542, dMSC-sEVs failed to upregulate pSmad2/3 expression (Fig. 8c). Furthermore, we also observed that SB431542 partially abolished the decrease of SA-β-gal activity induced by dMSC-sEVs (Fig. 8d, e), which represented that SB431542 partly blocked the protective effects of dMSC-sEVs on senescent fibroblasts. From the above results, we concluded that dMSC-sEVs have the protective effects on senescent fibroblasts by suppressing RAGE pathway and activating Smad pathway.

### dMSC-sEVs enhanced diabetic chronic wound healing

**Fig. 9:**
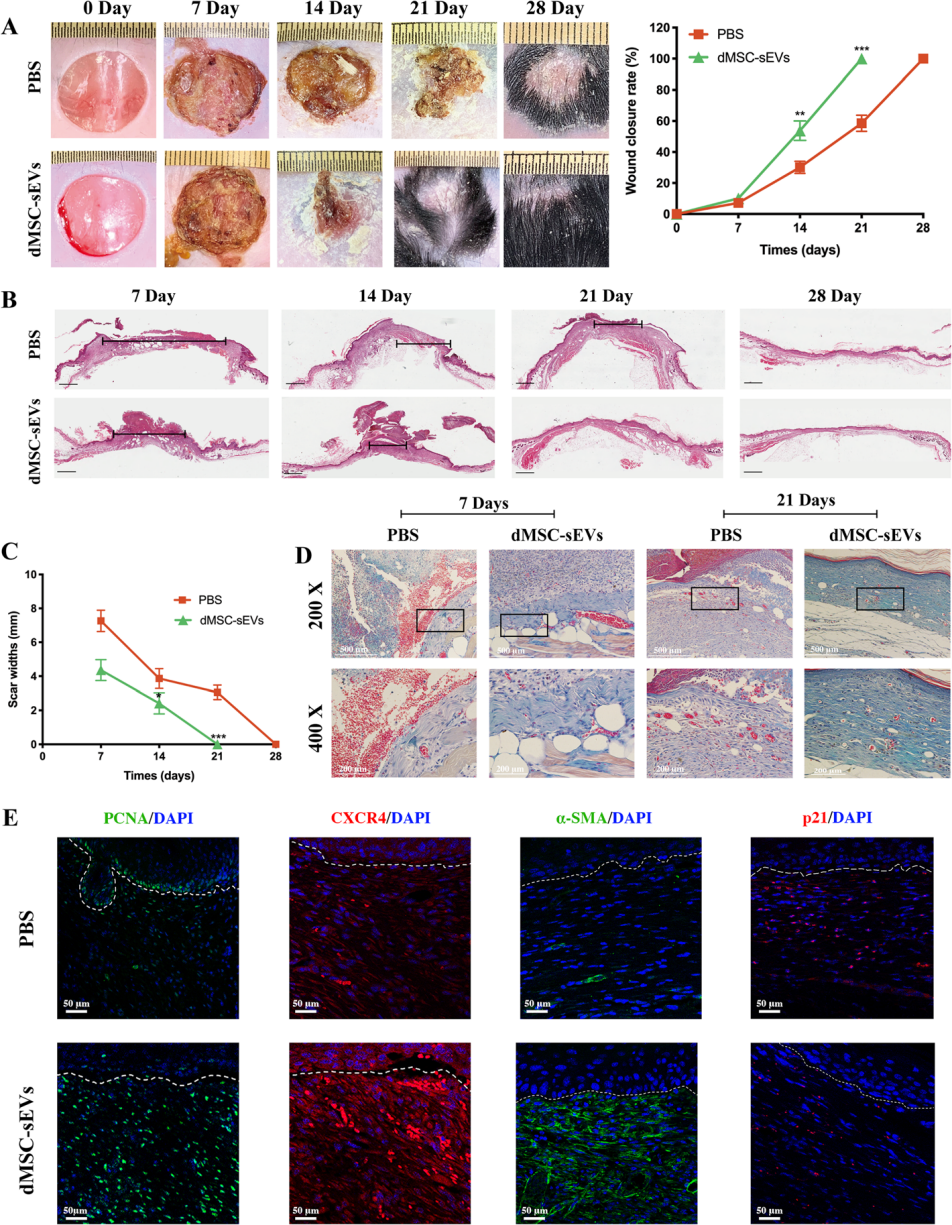
dMSC-sEVs enhanced diabetic wound healing. **a** Representative photographs of healing progression from day 0 to day 28. The statistical analysis of wound area in the dMSC-sEVs and PBS groups (*n* = 5). **b**, **c** Representative photographs of scar widths and the statistical analysis of the scar widths (*n* = 5), scale bar = 1 mm. **d** Masson’s trichrome staining at day 7 and day 21 post- wounding. The collagen fiber was stained in blue. × 200, scale bar = 500 μm. × 400, scale bar = 200 μm. **e** Representative immunofluorescence images of PCNA, CXCR4, α-SMA, and p21 staining. Scale bar = 50 μm. *p < 0.05, **p < 0.01, ***p < 0.001

Full-thickness wounds were made to evaluate the effects of the dMSC-sEVs on wound healing of diabetic mice. Gross observation of dorsal wounds showed that a reduced wound area in the dMSC-sEV group was qualitatively visible (Fig. 9a). The wound areas were measured on 0, 7, 14, 21, and 28 days after wounding. Mice treated with dMSC-sEVs displayed greater wound closure than observed in the PBS groups at days 14 and 21 post-wounding. The narrowest scar widths were observed at day 14 post-wounding (dMSC-sEV group, 2.41 ± 0.24 mm; PBS group, 3.87 ± 0.60 mm, *p* < 0.05) (Fig. 9b, c). Moreover, a larger and better-organized collagen deposition was observed in dMSC-sEV-treated wounds (Fig. 9d). The data indicated that dMSC-sEVs significantly accelerated collagen deposition and thus promoted wound healing.

Immunofluorescence staining for the expression of PCNA, CXCR4, α-SMA, and p21 was performed to visualize the effects of dMSC-sEVs on fibroblast proliferation, migration, differentiation, and senescence in vivo at day 7 post-wounding (Fig. 9e). The results revealed that few of PCNA-positive cells were observed in the PBS group, while a large number of proliferating cells appeared in the wounds in the dMSC-sEV group. CXCR4 expression was notably upregulated in the dMSC-sEV group rather than that in the PBS group, representing an enhanced migration ability of fibroblasts. In addition, dMSC-sEVs also improved the expression of α-SMA. Furthermore, a large number of senescent HDFs which positively expressed p21 were observed at wound beds in the PBS group, while the aged HDFs were barely identified in the dMSC-sEV group. These results indicate that dMSC-sEVs could enhance fibroblast proliferation, migration, and differentiation abilities and improve fibroblast senescent state in vivo, thereby accelerating wound healing in diabetic mouse.

## Discussion

In the present study, we reported that dMSC-sEVs improved the function of HG-induced HDFs and thereby accelerated diabetic wound repair. HDF senescent model was induced by HG with the concentration of 35 mM. dMSC-CM containing sEVs enhanced fibroblast proliferation and migration abilities. To further confirm the role of dMSC-sEVs, dMSC-sEVs were isolated and applied to treat the HG-induced fibroblasts. The results showed that dMSC-sEVs improved fibroblast senescent state and increased the proliferation, migration, and differentiation abilities of HDFs. The possible mechanisms of improved fibroblast function were related with the depression of RAGE pathway and activation of Smad pathway. Furthermore, dMSC-sEVs could enhance diabetic wound healing with improved functional states of fibroblasts. These suggest that dMSC-sEVs may be an effective therapy for diabetic wounds.

During the wound healing process, HDFs are attracted from the edge of the wound. At the stage of new tissue formation, some of HDFs differentiate into myofibroblasts which can produce ECM, mainly in the form of collagen [29]. In diabetic patients, HG microenvironment impairs cellular function. Accumulating evidence suggests that the HDFs from diabetic mice and rats exhibit a marked reduction in migratory ability compared with those from normal control mice [18, 30]. In this study, we found that HG with the concentration of 35 mM decreased the proliferation and migration abilities of fibroblasts and accelerated fibroblast senescence. In agreement with our results, previous studies demonstrated that prolonged HG induced senescence in HDFs through activation of p21 and p16 in a ROS-dependent manner, which further delayed the viability and migration in HDFs [20].

sEVs play an indispensable role in cell-to-cell communication [8] because they carry nucleic acids, lipids, and proteins. Biological characteristics and functions of sEVs suggest their potential application for cell-free regeneration strategies, which may avoid the disadvantages of current stem cell transplantation techniques. More and more studies have demonstrated that sEVs are able to enhance wound healing [14]. Previously, Zhao et al. demonstrated that exosomal microRNAs derived from human amniotic epithelial cells accelerated wound healing by promoting the proliferation and migration of HDFs [31]. Besides, Dalirfardouei et al. found that exosomes derived from menstrual blood MSCs enhanced re-epithelialization in the diabetic wound mice [32]. In our experiments, dMSC-sEVs were isolated to treat the HG-induced fibroblasts. The result showed dMSC-sEVs not only promoted fibroblast proliferation, migration, and differentiation, but also improved fibroblast senescent state.

With the improvement of the senescent state of HG-induced fibroblasts, dMSC-sEVs may have effects on diabetic wound healing. To confirm our hypothesis, dMSC-sEVs were used to treat the wounds. Our results showed that dMSC-sEVs was able to promote diabetic wound healing with enhanced collagen deposition. Furthermore, we found that dMSC-sEVs can promote the fibroblast proliferation, migration, and differentiation and decrease the senescence of HDFs in vivo as evidenced by immunofluorescence staining of PCNA, CXCR4, α-SMA, and p21. PCNA is specially expressed during the S phase of the cell cycle and is a marker of cell proliferation [16]. CXCR4 is a cell migration marker [33]. Consistent with previous studies [34], our study found that dMSC-sEVs promoted CXCR4 expression. These suggest that sEVs may be an ideal option for patients who suffer from chronic wound. However, the mechanisms underlying the effects of sEVs have remained elusive.

AGEs have been found to accumulate in the tissues of animals with diabetes [35]. AGEs have been reported to interfere with matrix-cell interactions by altering the cross-linking of the extracellular matrix and impairing wound-healing [36]. It is well accepted that AGEs contribute to a variety of diabetes complications through the cross-linking of molecules in the extracellular matrix and by engaging RAGE [37]. High glucose environment upregulates expression of RAGE and then accumulates intracellular ROS generation [22]. The upregulation of RAGE signaling exaggerates cellular responses to cause tissue destruction [38]. In this study, the downregulation of RAGE and p21 RAS confirmed the antioxidant effects of dMSC-sEVs. Smad signaling pathway was reported to be involved in fibroblast differentiation [39]. Our results showed that dMSC-sEVs increased the expression of myofibroblast markers and pSmad2/3. Furthermore, pSmad2/3 inhibitor SB431542 damaged the protective effects of dMSC-sEVs on senescent fibroblasts. So we deduce that dMSC-sEVs ameliorate HDFs senescence status by both RAGE and Smad signaling pathway.

## Conclusion

In conclusion, our results showed that dMSC-sEVs can effectively promote diabetic wound healing in mice by enhancing fibroblast proliferation, migration, and differentiation abilities and improving fibroblast senescent state. The underlying mechanism may be by suppressing RAGE pathway and activating Smad pathway. Our findings suggest that dMSC-sEVs will be a promising therapeutic tool for diabetic wound healing.

## Data Availability

Not applicable.

## Abbreviations

HG: High-glucose
sEVs: Small extracellular vesicles
dMSC-sEVs: Small extracellular vesicles derived from decidua-derived mesenchymal stem cells
HDFs: Human dermal fibroblasts
dMSC-CM: Decidual-derived mesenchymal stem cell-conditioned medium
ECM: Extracellular matrix
AGEs: Advanced glycation end products
ROS: Generate reactive oxygen species
dMSCs: Decidual-derived mesenchymal stem cells
PMSCs: Human placenta mesenchymal stem cells
DMEM/F12: Dulbecco’s modified Eagle’s medium/F12
FBS: Fetal bovine serum
SA-β-gal staining: Senescence-associated β-galactosidase staining
TEM: Transmission electron microscopy
Grp94: Glucose-regulated protein 94
CCK-8: Cell counting kit-8 assay
PI: Propidium iodide
PBS: Phosphate buffer saline
DCFH-DA: 2′,7′-Dichlorodihydrofluorescein diacetate
Anti-pSmad2/3: Anti-phosphorylate Smad2/3
SEM: Standard error of mean
NTA: Nanoparticle tracking analyzer

## References

[1] Clinton A, Carter T (2015). Chronic wound biofilms: pathogenesis and potential therapies. Lab Med.

[2] Lefrancois T (2017). Evidence based review of literature on detriments to healing of diabetic foot ulcers. Foot Ankle Surg.

[3] Dong L (2017). A conditioned medium of umbilical cord mesenchymal stem cells overexpressing Wnt7a promotes wound repair and regeneration of hair follicles in mice. Stem Cells Int.

[4] Darby I, Hewitson T (2007). Fibroblast differentiation in wound healing and fibrosis. Int Rev Cytol.

[5] Wertheimer E (2001). The regulation of skin proliferation and differentiation in the IR null mouse: implications for skin complications of diabetes. Endocrinology..

[6] Huebschmann A (2006). Diabetes and advanced glycoxidation end products. Diabetes Care.

[7] Chen L (2008). Paracrine factors of mesenchymal stem cells recruit macrophages and endothelial lineage cells and enhance wound healing. PLoS One.

[8] Panfoli I (2018). Microvesicles as promising biological tools for diagnosis and therapy. Expert Rev Proteomics.

[9] Muralidharan-Chari V (2010). Microvesicles: mediators of extracellular communication during cancer progression. J Cell Sci.

[10] Ma T (2019). Adipose mesenchymal stem cell-derived exosomes promote cell proliferation, migration, and inhibit cell apoptosis via Wnt/β-catenin signaling in cutaneous wound healing. J Cell Biochem.

[11] Ballen KK (2013). Umbilical cord blood transplantation: the first 25 years and beyond. Blood..

[12] Komaki M (2017). Exosomes of human placenta-derived mesenchymal stem cells stimulate angiogenesis. Stem Cell Res Ther.

[13] Du W (2017). Enhanced proangiogenic potential of mesenchymal stem cell-derived exosomes stimulated by a nitric oxide releasing polymer. Biomaterials..

[14] Hu L (2016). Exosomes derived from human adipose mensenchymal stem cells accelerates cutaneous wound healing via optimizing the characteristics of fibroblasts. Sci Rep.

[15] Théry C (2006). Isolation and characterization of exosomes from cell culture supernatants and biological fluids. Curr Protoc Cell Biol.

[16] Wang F (2020). Akermanite bioceramic enhances wound healing with accelerated reepithelialization by promoting proliferation, migration, and stemness of epidermal cells. Wound Repair Regen.

[17] Zhao B (2017). Exosomes derived from human amniotic epithelial cells accelerate wound healing and inhibit scar formation. J Mol Histol.

[18] Lamers ML (2011). High glucose-mediated oxidative stress impairs cell migration. PLoS One.

[19] Dimri GP (1995). A biomarker that identifies senescent human cells in culture and in aging skin in vivo. Proc Natl Acad Sci U S A.

[20] Li M (2017). Umbilical cord-derived mesenchymal stromal cell-conditioned medium exerts in vitro antiaging effects in human fibroblasts. Cytotherapy..

[21] Sharpless NE, Sherr CJ (2015). Forging a signature of in vivo senescence. Nat Rev Cancer.

[22] Li, B, et al., Regenerative and protective effects of calcium silicate on senescent fibroblasts induced by high glucose, Wound Repair Regen. 2020. 10.1111/wrr.12794. 10.1111/wrr.12794 31943524

[23] Prodinger C (2017). Current and future perspectives of stem cell therapy in dermatology. Ann Dermatol.

[24] Marfia G (2015). Mesenchymal stem cells: potential for therapy and treatment of chronic non-healing skin wounds. Organogenesis..

[25] Joanna K (2016). Proteomic comparison defines novel markers to characterize heterogeneous populations of extracellular vesicle subtypes. PNAS..

[26] Chen X (2010). Mechanistic study of endogenous skin lesions in diabetic rats. Exp Dermatol.

[27] Loughlin D, Artlett C (2009). 3-Deoxyglucosone-collagen alters human dermal fibroblast migration and adhesion: implications for impaired wound healing in patients with diabetes. Wound Repair Regen.

[28] Goova M (2001). Blockade of receptor for advanced glycation end-products restores effective wound healing in diabetic mice. Am J Pathol.

[29] Mathieu D (2006). Non-healing wounds.

[30] Lerman O (2003). Cellular dysfunction in the diabetic fibroblast: impairment in migration, vascular endothelial growth factor production, and response to hypoxia. Am J Pathol.

[31] Zhao B (2018). Exosomal microRNAs derived from human amniotic epithelial cells accelerate wound healing by promoting the proliferation and migration of fibroblasts. Stem Cells Int.

[32] Dalirfardouei R (2019). Promising effects of exosomes isolated from menstrual blood-derived mesenchymal stem cell on wound-healing process in diabetic mouse model. J Tissue Eng Regen Med.

[33] Van S (2019). Human umbilical cord blood mesenchymal stem cells expansion via human fibroblast-derived matrix and their potentials toward regenerative application. Cell Tissue Res.

[34] Rembe J (2018). Effects of vitamin B complex and vitamin C on human skin cells: is the perceived effect measurable?. Adv Skin Wound Care.

[35] Chang P (2013). Progression of periodontal destruction and the roles of advanced glycation end products in experimental diabetes. J Periodontol.

[36] Mealey B (2006). Diabetes mellitus and periodontal diseases. J Periodontol.

[37] Goh S, Cooper M (2008). Clinical review: the role of advanced glycation end products in progression and complications of diabetes. J Clin Endocrinol Metab.

[38] Sparvero L (2009). RAGE (receptor for advanced glycation endproducts), RAGE ligands, and their role in cancer and inflammation. J Transl Med.

[39] Zhou Y (2018). Induced pluripotent stem cell-conditioned medium suppresses pulmonary fibroblast-to-myofibroblast differentiation via the inhibition of TGF-β1/Smad pathway. Int J Mol Med.

